# Controlled-release fertilizer affects leaf nitrogen allocation and photosynthesis to improve nitrogen use efficiency and yield in the sunflower field

**DOI:** 10.3389/fpls.2025.1622766

**Published:** 2025-07-07

**Authors:** Wenhao Ren, Xianyue Li, Tingxi Liu, Ning Chen, Maoxin Xin, Qian Qi, Bin Liu

**Affiliations:** ^1^ National Key Laboratory of Ecological Environment of Arid Region Water Engineering, Inner Mongolia Agricultural University, Huhhot, China; ^2^ Collaborative Innovation Center for Integrated Management of Water Resources and Water Environment in the Inner Mongolia Reaches of the Yellow River, Hohhot, China; ^3^ Research and Development of Efficient Water-saving Technology and Equipment and Research Engineering Center of Soil and Water Environment Effect in Arid Area of Inner Mongolia Autonomous Region, Hohhot, China

**Keywords:** sunflower, photosynthetic-nitrogen use efficiency, controlled-release fertilizer, leaf nitrogen allocation, sustainable agricultural development

## Abstract

**Introduction:**

Nitrogen (N) can significantly affect the photosynthetic rate (Pn) of plants. Under traditional nitrogen fertilization (TNF) or inappropriate nitrogen application, leaf N is often redistributed to support the seed protein accumulation rather than the photosynthesis in the later stages of crop growth. Controlled-release fertilizers (CRF) have been reported to effectively reduce the nitrogen loss by matching the release pattern with crop N demand, thus increasing the yield. However, the changes in N allocation to enhance the photosynthesis under CRF have rarely been addressed.

**Methods:**

A two-year field experiment was conducted in the Hetao Irrigation District, Inner Mongolia, China from 2019 to 2020 to evaluate the effects of different fertilization strategies on soil NO_3_-N concentration, leaf nitrogen content, photosynthetic characteristics, yield, and nitrogen use efficiency (NUE) in sunflowers. The treatments included the CRF application rates of 135, 225, and 315 kg/ha (CRF_135_, CRF_225_, and CRF_315_), and that of TNF at 225 kg/ha (TNF_225_).

**Results:**

The results demonstrated that applying CRF at an appropriate rate maintained a high level of photosynthetic nitrogen content in the leaves during the later growth stages. This rate ensured a suitable soil NO_3_-N concentration (SNC), resulting in a 76.10% higher proportion of photosynthetic nitrogen (N_psn_) than TNF at the same rate, significantly enhancing the photosynthetic nitrogen efficiency (PNUE) and highlighting the crucial role of nitrogen management in improving the crop productivity and NUE. Additionally, at CRF_225_, the net photosynthesis (Pn), stomatal conductance (Gs), and intercellular CO_2_ concentration (Ci) at maturity increased by 32.80%, 96.16%, and 13.56%, respectively, compared to TNF, leading to an 11.84% improvement in yield and a 9.70% increase in NUE.

**Discussion:**

The correlation analysis confirmed a strong positive relationship between leaf N redistribution and photosynthetic efficiency, demonstrating the potential of CRF to improve the photosynthetic efficiency, optimize the N management, and promote the environmental sustainability in sunflower cultivation.

## 1 Introduction

The global demand for food has been expected to surge as the population will approach 9.7 billion by 2050 (ECONOMIC, U.N.D.F and AFFAIRS, S, 2023), presenting an urgent challenge to enhance the crop production efficiency and ensure the sustainable management (Li et al., 2016; Liu et al., 2020). The excessive and improperly managed nitrogen fertilizers not only elevate production costs but also lead to adverse environmental impacts, including water body eutrophication, soil degradation, and greenhouse gas emissions (Penuelas and Sardans, 2022; Zhao et al., 2024). Extensive field research has demonstrated that optimizing nitrogen fertilizer application patterns can produce more grains with less nitrogen. Specifically, the yields for wheat, corn, and rice have increased by 10% to 19%, while the nitrogen fertilizer applications have decreased by 15% to 19%, resulting in a 32–46% improvement in NUE and a 40% reduction in nitrogen surplus (Ren et al., 2022b). Furthermore, a comprehensive review of over 8,000 studies across multiple countries revealed that CRF significantly improved the crop yield by 5.1%, the farmer profitability by 8.2%, and the total nitrogen uptake by crops by 7.1%, while substantially reducing the environmental pollution, including the greenhouse gas emissions by 3.6% to 18.6% and the nitrogen losses by 32.6% to 49.1%, compared to the traditional fertilizers (Zhang et al., 2024). Thus, optimizing fertilization strategies and selecting appropriate fertilizer types are crucial for increasing NUE, enhancing crop yield, and mitigating environmental pollution risks.

The nitrogen fertilizer management is pivotal in modern agriculture as it directly influences the crop yield and ecosystem health (Huang et al., 2024; Seleiman et al., 2020; Wan et al., 2021). Represented by urea, traditional nitrogen fertilizers (TNF) can pose significant environmental and crop health risks when inappropriately applied (Carlson et al., 2016; Stolarski et al., 2017; Wang et al., 2022c). Although TNF can quickly enhance the crop nutrition, its high loss rates and low NUE contribute to increased production costs and environmental problems, such as soil acidification, groundwater pollution, and greenhouse gas emissions (Walling and Vaneeckhaute, 2020; Wang et al., 2022b). The nutrient losses from N fertilizers at approximately 50% highlight the inefficiencies in fertilizer use (Bindraban et al., 2020; Coskun et al., 2017). CRF designed with specific coating technologies to regulate nitrogen release offers a solution by aligning nutrient delivery with crop growth needs and absorption patterns (Hou et al., 2024; Salvagiotti et al., 2008; Sim et al., 2021). This approach not only improves the nitrogen utilization and crop nutrition (Cao et al., 2021; Vejan et al., 2021), but also reduces the environmental impact and supports healthy, stable crop growth, improving yield and quality (Hou et al., 2023; Li et al., 2020; Zhang et al., 2023). Despite evidence that CRF can enhance the nitrogen utilization efficiency and wheat yield by 32.49% and 18.20%, respectively, there is still potential for improvement (Ma et al., 2023). Sunflower is a major oilseed crop with high adaptability to arid and semi-arid climates (Ebrahimian et al., 2019; García-López et al., 2016). In northern China, particularly in regions like the Hetao Irrigation District, sunflower is widely cultivated due to its drought tolerance, relatively low water demand, and economic value (He and Liu, 2024; Ren et al., 2018). Compared to cereal crops, sunflower exhibits distinct nitrogen uptake dynamics and biomass partitioning patterns, making it a suitable model crop for studying NUE and the effectiveness of CRF under variable water-nitrogen conditions (Li et al., 2022; Ren et al., 2018). This is due to the need for a better understanding of CRF release dynamics and nitrogen application management. Therefore, further exploration of CRF’s role in the nitrogen release, crop responses, and environmental impacts is crucial for advancing agricultural practices towards greater efficiency and sustainability.

In exploring the sustainable optimization strategies for crop production, enhancing the photosynthetic efficiency is crucial, as it can directly affect the crop biomass accumulation and final yield (Mahmood et al., 2023; Wang et al., 2022a; Wu et al., 2019). The nitrogen supply can significantly influence photosynthesis by affecting the leaf structure and internal nitrogen distribution. The nitrogen deficiency can reduce photosynthesis, leaf area, and the lifespan of green leaves, thereby affecting the plant productivity (Liao et al., 2022; Nasar et al., 2022; Zhang et al., 2025). The nitrogen in crop leaves is categorized into four main types: photosynthetic nitrogen, respiratory nitrogen, storage nitrogen, and structural nitrogen (Ali et al., 2016; Dai et al., 2024; Liu et al., 2018b; Sun et al., 2020). The photosynthetic nitrogen can be further divided into three systems: the carboxylation system (N_cb_), which includes proteins such as Rubisco involved in the Calvin cycle (Dai et al., 2024; Qiang et al., 2023); the electron transport system (N_et_) referring to the proteins involved in electron transfer (Nolfi-Donegan et al., 2020; Yoshida and Hisabori, 2024); and the light-harvesting system (N_cl_) that consists of the proteins in photosystems I and II and other light-harvesting pigment-protein complexes (Grouneva et al., 2016; Liu et al., 2018b). The non-photosynthetic nitrogen is classified into respiratory nitrogen (N_resp_) that includes the respiratory enzymes in the mitochondrial matrix (Hou et al., 2019); storage nitrogen (N_store_) stored in tissues and does not participate in metabolic processes; and structural nitrogen (N_str_) primarily adopted to build cell walls and nucleic acids (Hu et al., 2023a). The distribution patterns of nitrogen components among various crops lead to the differences in species-specific net photosynthetic rates and NUE (Gu, 2023; Li et al., 2019; Liu et al., 2018a; Tian et al., 2022). Proper nitrogen distribution among the different functions is essential for the crop growth and photosynthetic efficiency (Gao et al., 2022; Hu et al., 2023a; Jia et al., 2021). Research has indicated that CRF can optimize the nitrogen distribution in the soil with their slow-release properties aligning with the physiological nitrogen needs of crops to ensure an adequate supply (Trenkel, 2021; Vejan et al., 2021). However, there is limited research on how CRF affects nitrogen distribution in crop leaves to enhance photosynthesis-related parameters, such as photosynthetic rate (Pn), stomatal conductance (Gs), and intercellular CO_2_ concentration (Ci). An optimized nitrogen management strategy using CRF can create a healthier and more efficient photosynthetic environment, significantly improving the sunflower growth rate and yield. Additionally, the CRF application can contribute to the improved soil health and ecosystem services by reducing nitrogen loss (Ma et al., 2023; Xiao et al., 2019), protecting soil structure, and preserving microbial diversity (Gao et al., 2022; Trenkel, 2021), all of which support the sustained and efficient photosynthesis (Gao et al., 2024; Iriti et al., 2019; Radušienė et al., 2019). Therefore, it is crucial to further investigate the effect of varying soil NO_3_-N concentrations throughout the crop growth period under different CRF nitrogen application conditions. This approach seeks to enhance photosynthesis by altering the nitrogen distribution among different functions in crop leaves, ultimately improving NUE and increasing crop yield.

This study aimed to systematically assess the impact of CRF on sunflower growth and nitrogen management, addressing the gaps in existing research and offering actionable recommendations for agricultural practice. The specific objectives were: (1) to evaluate the physiological mechanisms of photosynthetic nitrogen use efficiency (PNUE) in the sunflowers influenced by CRF by analyzing the optimized distribution of nitrogen in sunflower leaves under suitable nitrogen application conditions; (2) to explore how CRF optimized the photosynthesis in sunflowers by measuring the key parameters such as Pn, Gs, and Ci, thereby enhancing the photosynthetic efficiency; (3) to analyze the impact of CRF on the nitrogen accumulation and distribution in the sunflower plants and assess how this mechanism affected the crop yield; and (4) to compare CRF with TNF to discuss the advantages of CRF in improving NUE, and to determine the optimal nitrogen application rate for CRF treatment to achieve the environmentally friendly and economically efficient nitrogen management strategies.

## 2 Materials and methods

### 2.1 Experimental materials and design

**Figure 1:**
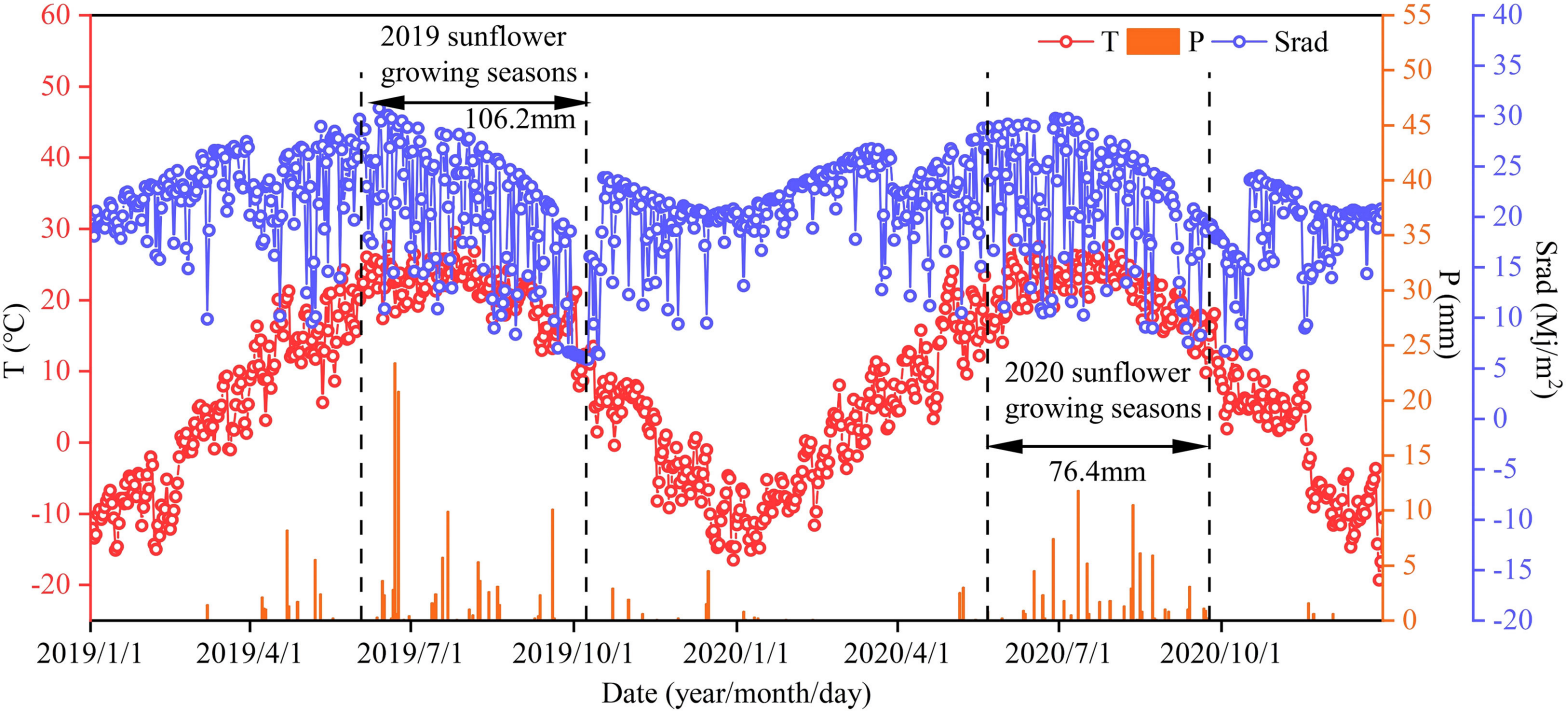
Average temperature (T), precipitation (P) and solar radiation (Srad) during the sunflower growing seasons in 2019 and 2020.

This study was conducted in the Ganzhaomiao Town experimental field (40°47’54”N, 107°16’42”E), Linhe District, Bayannaoer City, Inner Mongolia situated in a mid-temperate semi-arid continental climate zone. The field features the sandy loam soil ideal for sunflower growth (USDA) with the average bulk density of 1.40 g/cm^3^. The soil nutrient testing of the 0–100 cm layer before sowing in spring 2019 revealed the organic matter content of 6.19 g/kg, the available nitrogen of 34.43 mg/kg, the available phosphorus of 1.84 mg/kg, the available potassium of 113.04 mg/kg, and a soil pH of 8.5. The total rainfall during the growing seasons of 2019 and 2020 was 106.2 and 76.4 mm, respectively. All the climatic data were provided by an automatic weather station (Onset Computer Inc., U30, Hobo, USA) located in the experimental field (**Figure 1**).

The sunflowers (Xinjiang Sanrui, SH361) were sown on June 3, 2019, and May 22, 2020, and harvested on October 8 and September 24, respectively. A conventional ridge–furrow planting system following a “one-film-two-rows” configuration was adopted. The study included four treatments: CRF at 135, 225, and 315 kg/ha, and TNF at 225 kg/ha. CRF was applied as a base fertilizer in a single application prior to sowing. TNF comprising the diammonium phosphate (18% N, 46% P_2_O_5_) as the base fertilizer and urea (46% N) as the top dressing was applied with the diammonium phosphate (1/3 N) before sowing, and the urea (2/3 N) was manually spread before irrigation at the budding stage. The furrow irrigation was performed on July 14 in both 2019 and 2020, with an irrigation amount of 120 mm each. Standard management measures were employed to control diseases, pests, and weeds.

The CRF used in this s experiment was the sixth-generation product developed by Tianjin Luyang Fertilizer Co., Ltd., with a nutrient composition of N:P:K = 28:12:10. It employs a bioactive double-membrane dual-control coating technology that enables precise regulation of nutrient release. The nutrient release rate in static water at 25°C is ≤5% within 24 hours, ≤15% within 7 days, and ≤65% within 28 days. The cumulative nutrient release over the release period is ≥80%. At an average soil temperature of 25°C, the nutrient release period is 70 d; at 15°C, it is 100 d; and at 10°C, it is 170 d.

### 2.2 Sampling and measurements

#### 2.2.1 Soil NO_3_-N concentration

The soil NO_3_-N concentration (SNC) was measured using the semi-micro Kjeldahl method (Bremner, 1965). The soil samples were collected with a soil auger (Beijing New Landmark Soil Equipment Co., Ltd., 0301, XDB, CHN) from various depths (0–10 cm, 10–20 cm, 20–30 cm, 30–40 cm, 40–50 cm, 50–60 cm, 60–80 cm, and 80–100 cm) in both the mulched area (soil beneath the plastic film between the planted rows) and non-mulched area (soil between adjacent film strips without plastic cover) within 10- to 15-day intervals. To ensure the accuracy, three samples were collected for each measurement. The collected soil was air-dried, ground, thoroughly mixed to achieve uniformity, and passed through a 1 mm sieve. For the SNC determination, 5 g of soil was mixed with 25 mL of a 2 mol L^-1^ potassium chloride solution, shaken, and filtered, while the NO_3_-N concentration was quantified using an ultraviolet spectrophotometer (Beijing General Instrument Co., Ltd., TU-1901, CHN).

#### 2.2.2 Photosynthetic characteristics

Three sunflower plants in each plot were selected and marked for the measurement of their photosynthetic characteristics at four stages: seedling (30 days after sowing (DAS), with 5–7 fully expanded leaves and 25–35 cm in height), budding (50 DAS, with visible apical flower buds and 12–16 leaves), flowering (70 DAS, with fully open capitulum and maximum leaf area development), and maturity (90 DAS, with top leaves still green and photosynthetically active). Measurements were performed nine times (three replicates, each repeated three times) using a portable photosynthesis system (Beijing, ECO Tech, Cpro T). The data collected included net photosynthetic rate (Pn, μmol CO_2_/m^2^/s), stomatal conductance (Gs, μmol H_2_O/m^2^/s), and intercellular CO_2_ concentration (Ci, μmol CO_2_/mol). Photosynthetically active radiation (PAR), CO_2_ concentration, flow rate, and leaf chamber temperature were set to 1700 μmol/m^2^/s, 380 μmol/mol, 500 μmol/s, and 30°C, respectively.

#### 2.2.3 Leaf area

During the seedling, budding, flowering, and maturity stages of sunflowers, the length and width of the leaves from the bottom to the top of the plants were measured. The calculation formula (Equation 1) is as follows:


(1) LA=∑Lenth×Width×0.75

Where LA (cm^2^) is leaf area, Lenth (cm) is length of fully expanded leaves, width is length of fully expanded leaves, 0.75 is an empirical coefficient.

#### 2.2.4 Dry matter accumulation and plant nitrogen uptake

Five sunflowers were randomly selected as the representative healthy specimens from both the diagonal and central areas of each plot using a five-point scale during their growth and development (Su et al., 2022). The specimens were divided into four parts: leaves, stems, seeds, and roots. The ground dry matter weight was determined by drying the samples in an oven at 105°C for 60 min, followed by drying at 80°C until a constant weight was achieved. The dried samples were subsequently ground into powder for the nitrogen content determination (Bremner, 1965). The samples were digested using H_2_SO_4_-H_2_O_2_, and the total nitrogen content was measured using a flow analyzer (China Ocean Energy Future Technology Group, K9860, China).

Sunflower four parts biomass (kg/ha) = plant four parts dry matter weight (kg) × the number of plants per hectare. The N concentration was expressed on a dry-weight basis, and total N uptake and accumulation were calculated as the product of concentration and dry weight.

#### 2.2.5 Yield

At harvest time, ten mature sunflower plants were consecutively selected from each experimental plot, and all seeds from their flower heads were collected. The seeds were air-dried to a moisture content of approximately 8% before measuring the total yield. Additionally, the hundred-seed weight and number of seeds per head were determined annually to assess the yield composition.

### 2.3 Determining the distribution of nitrogen among functions

To determine the distribution of nitrogen among various functions, this study employed a portable photosynthesis system (Ecotech Ecology Technology, Beijing, model Cpro T) to measure CO_2_ response curves (Pn-Ci). The light intensity and leaf chamber temperature were maintained at 1000 μmol/m^2^ and 25°C, respectively. The CO_2_ concentrations were set in a specified sequence: 400, 300, 200, 150, 100, 80, 50, 400, 600, 800, 1000, and 1200 μmol/mol, and at each concentration, Pn (photosynthetic rate) and Ci (intercellular CO_2_ concentration) were measured and used to plot the CO_2_ response curves. After the CO_2_ concentration stabilized, the corresponding Pn and Ci values were recorded. Additionally, the maximum carboxylation rate (Vc,max) and maximum electron transport rate (Jmax) were calculated using the Farquhar, von Caemmerer, and Berry (FvCB) (Farquhar et al., 1980) model and the R package “plantcophys” (Duursma, 2015). The proportions of nitrogen distributed among the different functions were then calculated using formulas from previous studies (Jordan and Ogren, 1984; Makino and Osmond, 1991; Niinemets and Tenhunen, 1997).


(2) $$N_{cb}=\frac{V_{c\max}N_{r}}{V_{cr}N_{area}}$$

Where *N*_cb_ represents the proportion of nitrogen allocated to the carboxylation system (Equation 2), N_r_ is the amount of nitrogen in Rubisco, assumed to be 0.16gN/(g Rubisco), and V_cr_ is the specific activity of Rubisco, assumed to be 20.5 μmol CO_2_/(g Rubisco)/s at 25°C.


(3) $$N_{et}=\frac{J_{\max}N_{b}}{J_{mc}N_{area}}$$

Where *N*_et_ represents the proportion of nitrogen in the electron transport components (Equation 3), N_b_ is the amount of nitrogen in cytochrome f, assumed to be 0.1240695 g N/(μmol cytochrome f), and J_mc_ is the capacity of electron transport per cytochrome f, set to 156 μmol electron/(μmol cytochrome f)/s.


(4) $$N_{cl}=\frac{chlorophyll\;content}{C_{b}N_{area}}$$

where *N*_cl_ represents the nitrogen distribution in the light-harvesting system (Equation 4); and *C*_b_ is the chlorophyll binding of the thylakoid protein complexes, assumed to be 2.75 mmol chlorophyll (g chlorophyll N). To determine the chlorophyll content, 0.1 g of leaf tissue (excluding the main veins) was soaked in 25 mL of 95% ethanol for 48 h. The absorbance was then measured at the wavelengths of 665 and 649 nm, and the chlorophyll content was calculated using the specified formula (Equation 5):


(5) $$Chlorophyll\;content=\frac{(20.2D_{645}+8.02D_{663})\times V}{1000\times W}$$

Where, V is the volume of the extraction solution (25ml), and W is the weight of the leaf tissue being measured (0.1g).


(6) $$N_{resp}=\frac{0.015V_{c,\max}}{33.69\times 0.522\times N_{area}}$$

(7) $$N_{store}=1-N_{psn}-N_{resp}-N_{str}$$

(8) $$N_{non-psn}=N_{store}+N_{resp}+N_{str}$$

(9) $$N_{psn}=N_{cb}+N_{et}+N_{cl}$$

Where, N_store_ represents the proportion of nitrogen allocated to storage, N_psn_ represents the distribution of photosynthetic nitrogen, and N_non-psn_ represents the non-photosynthetic components, which include N_store_, N_resp_, and N_str_. N_resp_ represents the distribution of respiratory nitrogen, N_str_ represents the distribution of structural nitrogen, and the photosynthetic components include N_cb_, N_et_, and N_cl_. N_str_, also known as SDS-insoluble N (N_in-SDS_), was measured as described previously (Equations 6–9) (Takashima et al., 2004).

### 2.4 Photosynthetic nitrogen use efficiency and nitrogen utilization efficiency

PNUE (µmol CO_2_/g/s) reflects the rate of CO_2_ assimilation per unit leaf area (Equation 10):


(10) $$PNUE=\frac{Pn}{LTNA/LA}=\frac{Pn}{N_{area}}$$

where *Pn* is the net photosynthetic rate, μmol CO_2_/m^2^/s; *LTNA* is the amount of total leaf N accumulation, mg/plant; *LA* is the total leaf area, cm^2^/plant; and *N_area_* denotes the leaf N content per unit area, mg/cm^2^.

In this study, three key nitrogen efficiency indices were adopted to comprehensively evaluate the sunflower’s efficiency in nitrogen uptake and utilization from the soil: Nitrogen Harvest Index (NHI), Partial Factor Productivity of Nitrogen (PFP), and NUE. NHI reflected the proportion of nitrogen in the harvested part of the crop and was applied as the important indicator of the crop’s ability to transfer and distribute nitrogen (Equation 11). PFP assessed the yield produced per unit of applied nitrogen and served as the indicator of nitrogen production efficiency (Equation 12). NUE provided the holistic view of the crop’s ability to utilize the available nitrogen resources (Equation 13).


(11) $$NHI=\frac{GN}{PNU}$$

(12) $$PFP=\frac{Yield}{F}$$

(13) $$NUE=\frac{Yield}{PNU}$$

Where *GN* is nitrogen content in grains; *PNU* is the uptake of N by the sunflower plant at maturity; *Yield* is the sunflower yield; F is the amount of applied N.

### 2.5 Statistical analysis

All data were processed and analyzed using Microsoft Excel 2021. Additionally, Origin software was employed to compare the mean values, calculate the least significant difference (LSD) at the 0.05 level, and create detailed graphical plots.

## 3 Results

### 3.1 Spatial and temporal distribution of soil NO_3_-N

**Figure 2:**
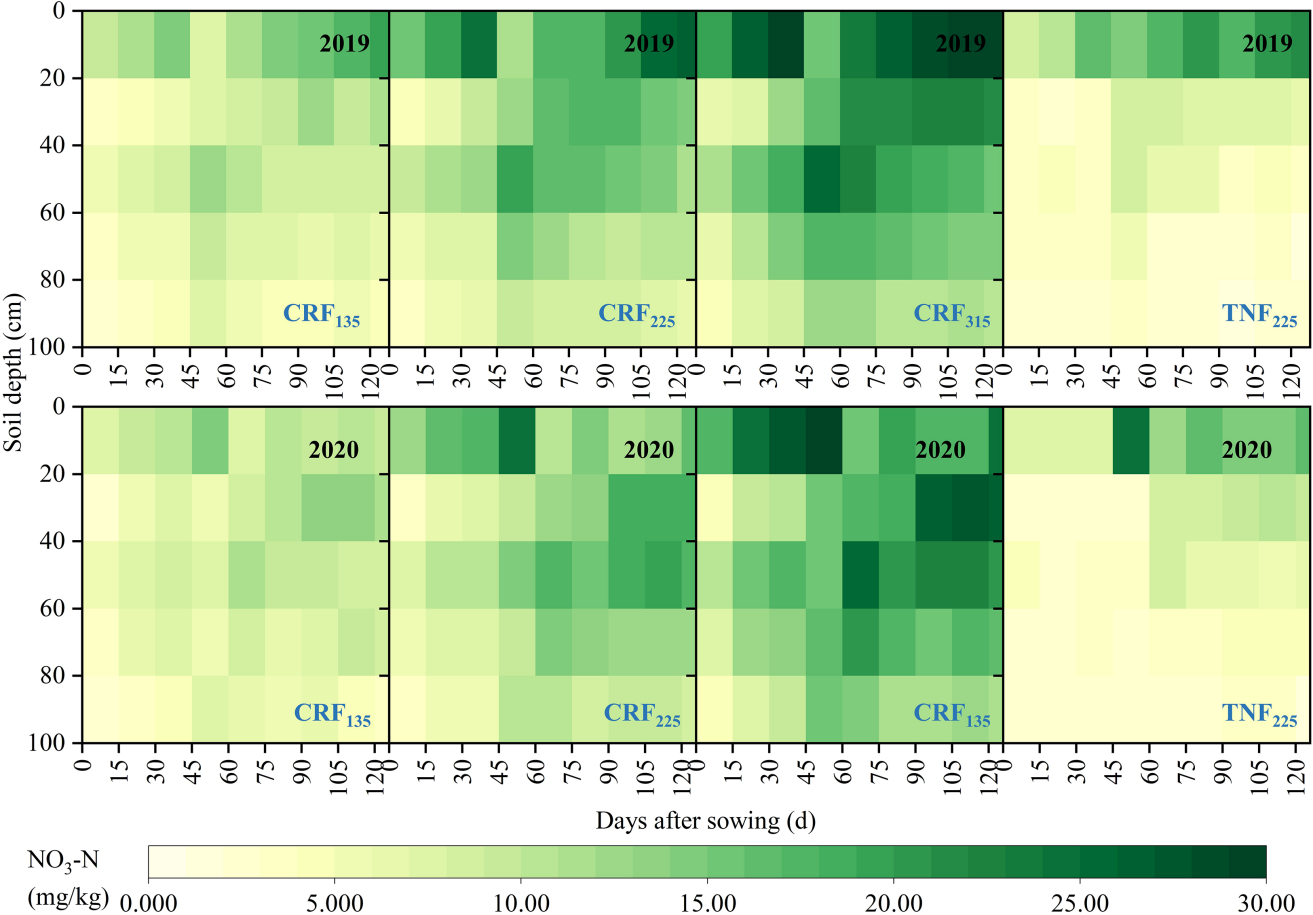
Soil NO_3_-N concentration (SNC) distribution during the growing season under the controlled-release fertilizer (CRF) three nitrogen application rates (subscripts 135, 225, and 315 kg/ha) and traditional nitrogen fertilizer (TNF) considering one nitrogen application rate (subscripts 225 kg/ha) from 2019 and 2020.

Different fertilization strategies significantly influenced the distribution of NO_3_-N in the soil profile during the sunflower growth (**Figure 2**). In the CRF treatment, the soil NO_3_-N concentration (SNC) gradually increased with the nitrogen application, whereas in the TNF treatment, SNC was higher only in the 0–20 cm soil layer. During the seedling and budding stages, the CRF_315_ and CRF_225_ treatments increased the average 0–40 cm SNC by 63.94% and 130.68%, respectively, compared to CRF_135_, and by 44.85% and 101.97%, respectively, during the flowering and maturity stages. Under the same nitrogen application conditions, the CRF treatments raised the 0–40 cm SNC by 52.79% during the seedling and budding stages and by 14.82% during the flowering and maturity stages compared with the TNF treatments. In the 40–100 cm layer, CRF_315_ and CRF_225_ increased SNC by 1.47 and 2.11 times during the seedling and budding stages, and by 1.47 and 2.19 times during the flowering and maturity stages, respectively, compared to CRF_135_. Under the same conditions, the CRF treatments increased SNC by 2.93 times compared to the TNF treatments.

### 3.2 Sunflower *N_area_* and PNUE under different nitrogen application conditions

**Figure 3:**
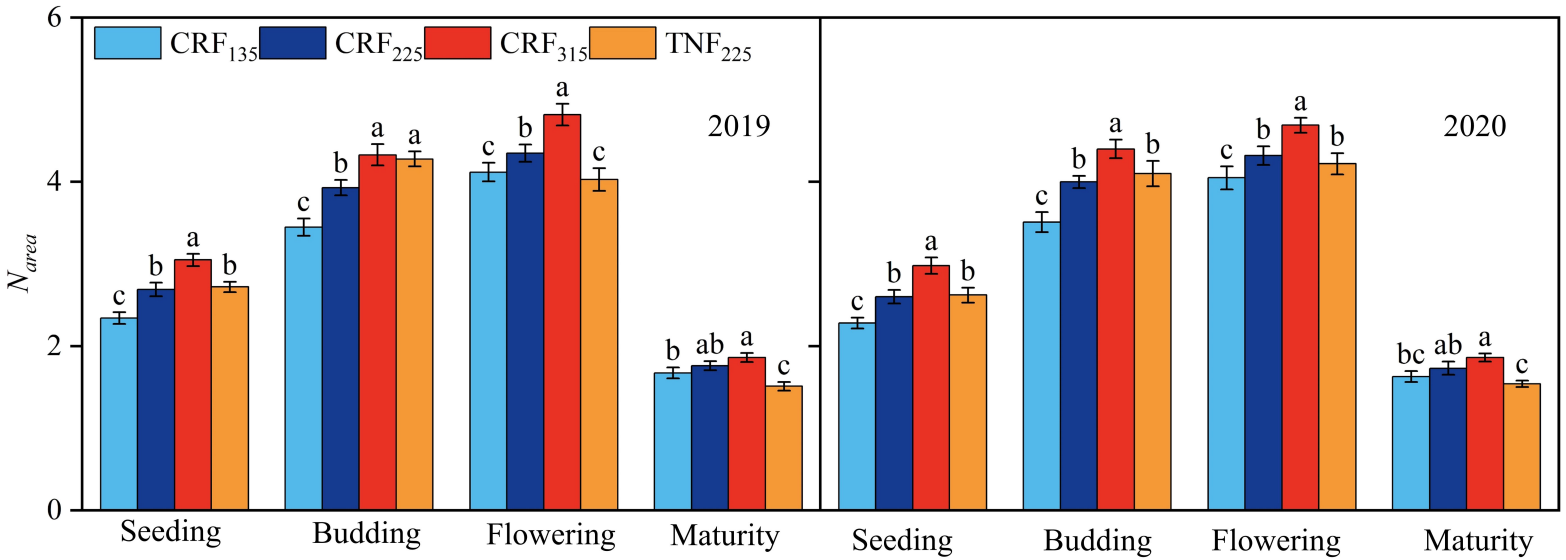
Nitrogen content per unit leaf area (*N_area_*) of sunflower at seeding, budding, flowering, and maturity growth stages in 2019 and 2020 under the controlled-release fertilizer (CRF) three nitrogen application rates (subscripts 135, 225, and 315 kg/ha) and traditional nitrogen fertilizer (TNF) considering one nitrogen application rate (subscripts 225 kg/ha).

**Figure 4:**
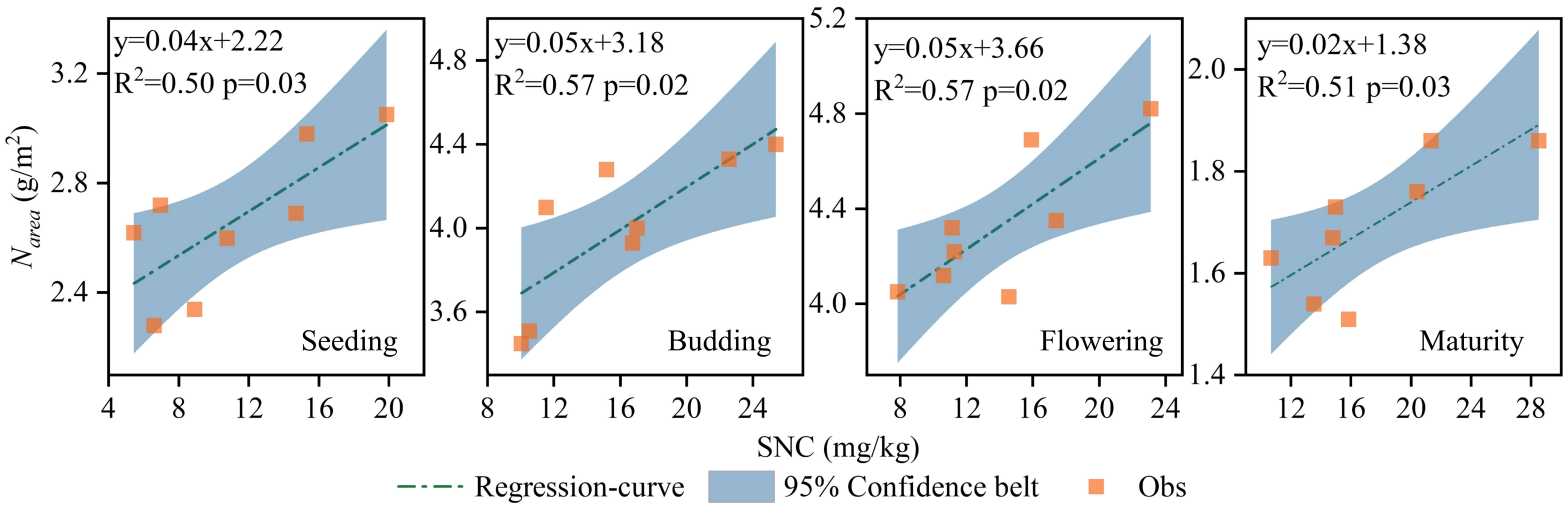
Correlation regression analysis between SNC and *N_area_* of sunflower at seeding, budding, flowering and maturity growth stages in 2019 and 2020 under the controlled-release fertilizer (CRF) three nitrogen application rates (subscripts 135, 225, and 315 kg/ha) and traditional nitrogen fertilizer (TNF) considering one nitrogen application rate (subscripts 225 kg/ha).

During the study periods of 2019 and 2020, the dry matter and nitrogen contents of sunflower leaves were measured at the seedling, budding, flowering, and maturity stages to calculate *N_area_* and PNUE. The fertilization treatments significantly affected both *N_area_* and PNUE, with the correlation regression analysis demonstrating a positive relationship between SNC and *N_area_* across all growth stages (**Figure 3**). Under CRF treatments, promoting the nitrogen application led to higher *N_area_* and PNUE, and both metrics were consistently higher under CRF than under the TNF treatments at the equivalent nitrogen levels. On average, *N_area_* in the CRF_315_ and CRF_225_ treatments was 21.27% and 10.08% higher, respectively, than in CRF_135_ over the two years. While *N_area_* in TNF_225_ was 10.48% higher than that in CRF_225_ during the seedling and budding stages, it was 9.62% lower during the flowering and maturity stages. *N_area_* increased initially but decreased later in the growth period, dropping by 60.81% (2.63 g/m^2^) at maturity compared to the flowering stage. The positive correlation between SNC and *N_area_* suggested that *N_area_* increased with the higher nitrate-nitrogen concentrations in the 0–40 cm soil layer throughout the sunflower growth cycle (**Figure 4**).

Regarding PNUE (**Table 1**), CRF treatments consistently outperformed TNF_225_ across all growth stages. At the maturity stage, CRF_315_ exhibited the highest PNUE, followed by CRF_225_ and CRF_135_, indicating a stronger capacity to sustain photosynthetic efficiency relative to leaf nitrogen content under high nitrogen input. However, when considering all stages together, CRF_225_ showed more stable and balanced performance, especially at the budding and flowering stages where it maintained relatively high PNUE with lower nitrogen input compared to CRF_315_. These findings suggest that CRF treatments, particularly CRF_225_, can effectively enhance PNUE and nitrogen utilization efficiency across critical growth stages of sunflower.

**Table 1:** Photosynthetic nitrogen use efficiency (PNUE) of sunflower at seeding, budding, flowering and maturity growth stages in 2019 and 2020 under the controlled-release fertilizer (CRF) three nitrogen application rates (subscripts 135, 225, and 315 kg/ha) and traditional nitrogen fertilizer (TNF) considering one nitrogen application rate (subscripts 225 kg/ha).

Years	Treatment	PNUE (μmol CO_2_/g/s)			
		Seeding	Budding	Flowering	Maturity
2019	CRF_135_	15.11 ± 0.98a	11.63 ± 0.57a	10.21 ± 0.3a	15.83 ± 1.02b
	CRF_225_	14.8 ± 0.74a	10.64 ± 0.27b	10.08 ± 0.39a	16.1 ± 0.43b
	CRF_315_	14.6 ± 0.64a	10.11 ± 0.24c	9.91 ± 0.4a	17.08 ± 0.5a
	TNF_225_	14.48 ± 0.5a	9.97 ± 0.29c	9.78 ± 0.39a	14.32 ± 0.62c
2020	CRF_135_	14.96 ± 0.49a	11.15 ± 0.29a	10.1 ± 0.4a	15.74 ± 0.55b
	CRF_225_	14.13 ± 0.58b	10.38 ± 0.3b	10.04 ± 0.28a	15.97 ± 0.58b
	CRF_315_	14.14 ± 0.46b	10.03 ± 0.3bc	9.71 ± 0.33ab	17.93 ± 0.46a
	TNF_225_	13.74 ± 0.68b	9.86 ± 0.37c	9.3 ± 0.45b	14.27 ± 0.46c

### 3.3 Proportion of N in leaves allocated to the photosynthetic system

**Figure 5:**
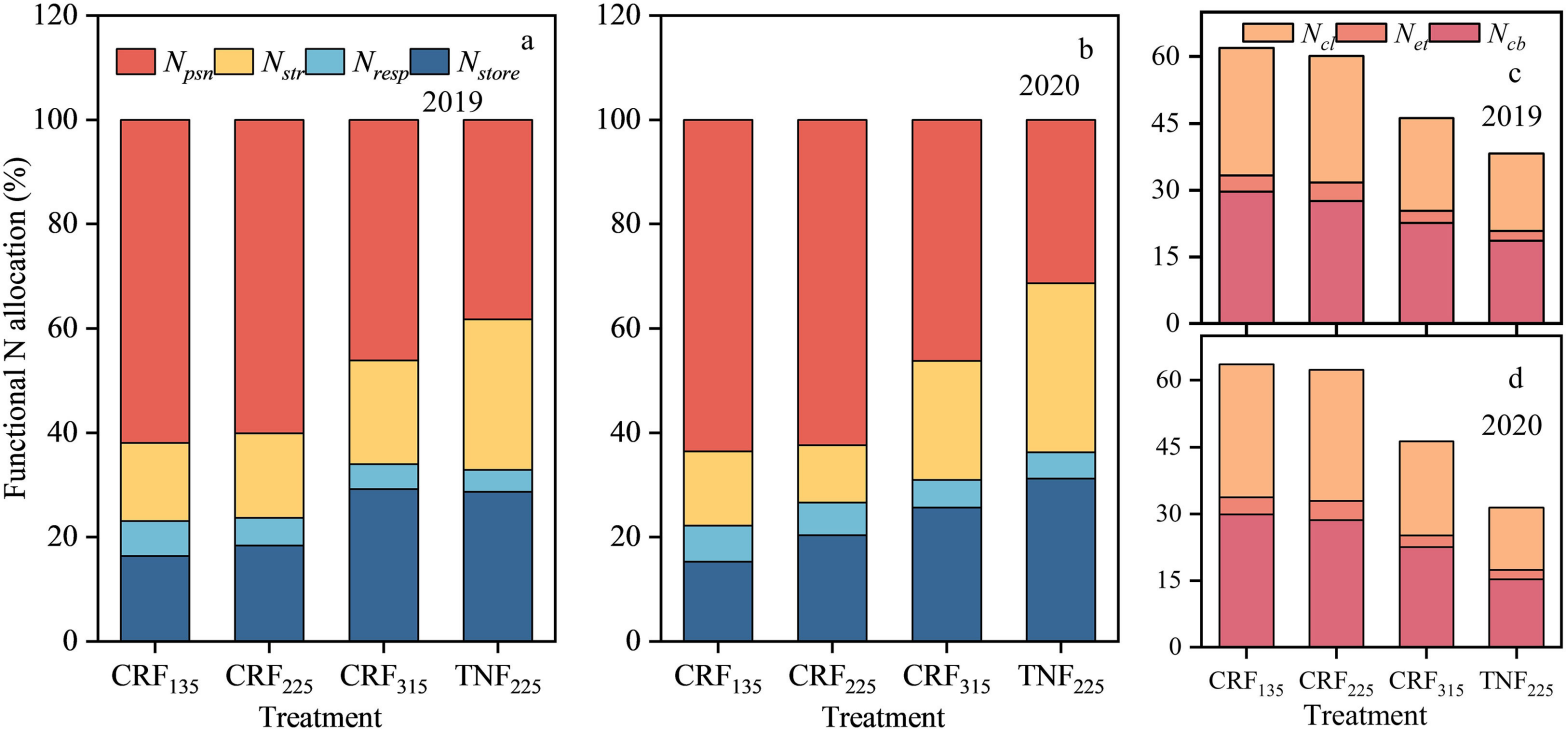
Distribution proportion of functional nitrogen (a and b), N_store_ means the distribution proportion of storage nitrogen, N_resp_ means the distribution proportion of respiratory nitrogen, N_str_ means the distribution proportion of structural nitrogen, N_psn_ means the distribution proportion of photosynthetic nitrogen; Distribution proportion of photosynthetic nitrogen (b and c), N_cb_ means the distribution proportion of carboxylation system, N_et_ means the distribution proportion of electron transfer component, N_cl_ means the distribution proportion of light harvesting system in 2019 and 2020 under the controlled-release fertilizer (CRF) three nitrogen application rates (subscripts 135, 225, and 315 kg/ha) and traditional nitrogen fertilizer (TNF) considering one nitrogen application rate (subscripts 225 kg/ha).

**Figure 6:**
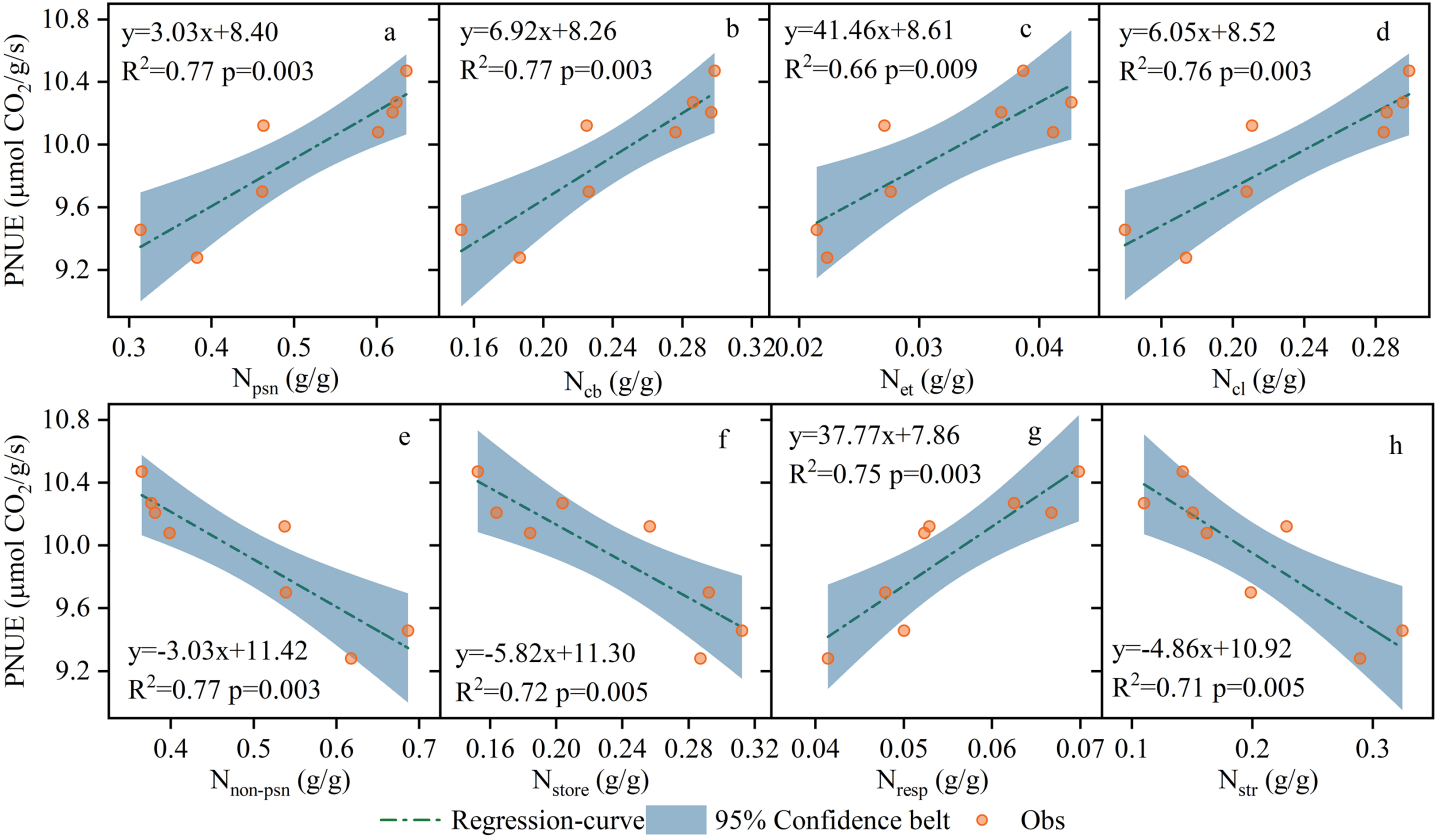
Correlation regression analysis between different N components and photosynthetic nitrogen use efficiency (PNUE), **(a)** Photosynthetic components (N_psn_). **(b)** carboxylation systems (N_cb_). **(c)** electron transfer components (N_et_). **(d)** light harvesting systems (Ncl). **(e)** non-photosynthetic components (N_non-psn_). **(f)** storage nitrogen (N_store_). **(g)** respiratory nitrogen (N_resp_). **(h)** structural nitrogen (N_str_) in 2019 and 2020 under the controlled-release fertilizer (CRF) three nitrogen application rates (subscripts 135, 225, and 315 kg/ha) and traditional nitrogen fertilizer (TNF) considering one nitrogen application rate (subscripts 225 kg/ha).

The proportion of N allocated to the photosynthetic system in the leaves was higher under CRF_225_ conditions. During the two-year experiment under CRF_135_, the proportions of N_store_, N_resp_, N_str_, and N_psn_ were 15.82%, 6.83%, 14.61%, and 62.75%, respectively (**Figure 5**). Compared to CRF_225_ and CRF_315_, Npsn under CRF_225_ increased by 2.43% and 35.84%, respectively, whereas N_non-psn_ decreased by 18.12% and 42.24%, respectively. At the 225 kg/ha nitrogen application rate with TNF, the proportions of N_store_, N_resp_, N_str_, and N_psn_ were 29.97%, 4.57%, 30.68%, and 34.79%, respectively, with N_psn_ under CRF_225_ being 76.10% higher than that under TNF_225_, and N_non-psn_ decreased by 40.60%. The correlation analysis revealed that N_psn_, N_cl_, N_et_, N_cb_, and N_resp_ were significantly positively correlated with PNUE, whereas N_store_ and N_str_ were significantly negatively correlated with PNUE (**Figure 6**).

**Figure 5** Distribution proportion of functional nitrogen (a and b), N_store_ means the distribution proportion of storage nitrogen, N_resp_ means the distribution proportion of respiratory nitrogen, N_str_ means the distribution proportion of structural nitrogen, N_psn_ means the distribution proportion of photosynthetic nitrogen; Distribution proportion of photosynthetic nitrogen (b and c), N_cb_ means the distribution proportion of carboxylation system, N_et_ means the distribution proportion of electron transfer component, N_cl_ means the distribution proportion of light harvesting system in 2019 and 2020 under the controlled-release fertilizer (CRF) three nitrogen application rates (subscripts 135, 225, and 315 kg/ha) and traditional nitrogen fertilizer (TNF) considering one nitrogen application rate (subscripts 225 kg/ha).

### 3.4 Photosynthetic characteristics of sunflowers under different CRF application conditions

**Figure 7:**
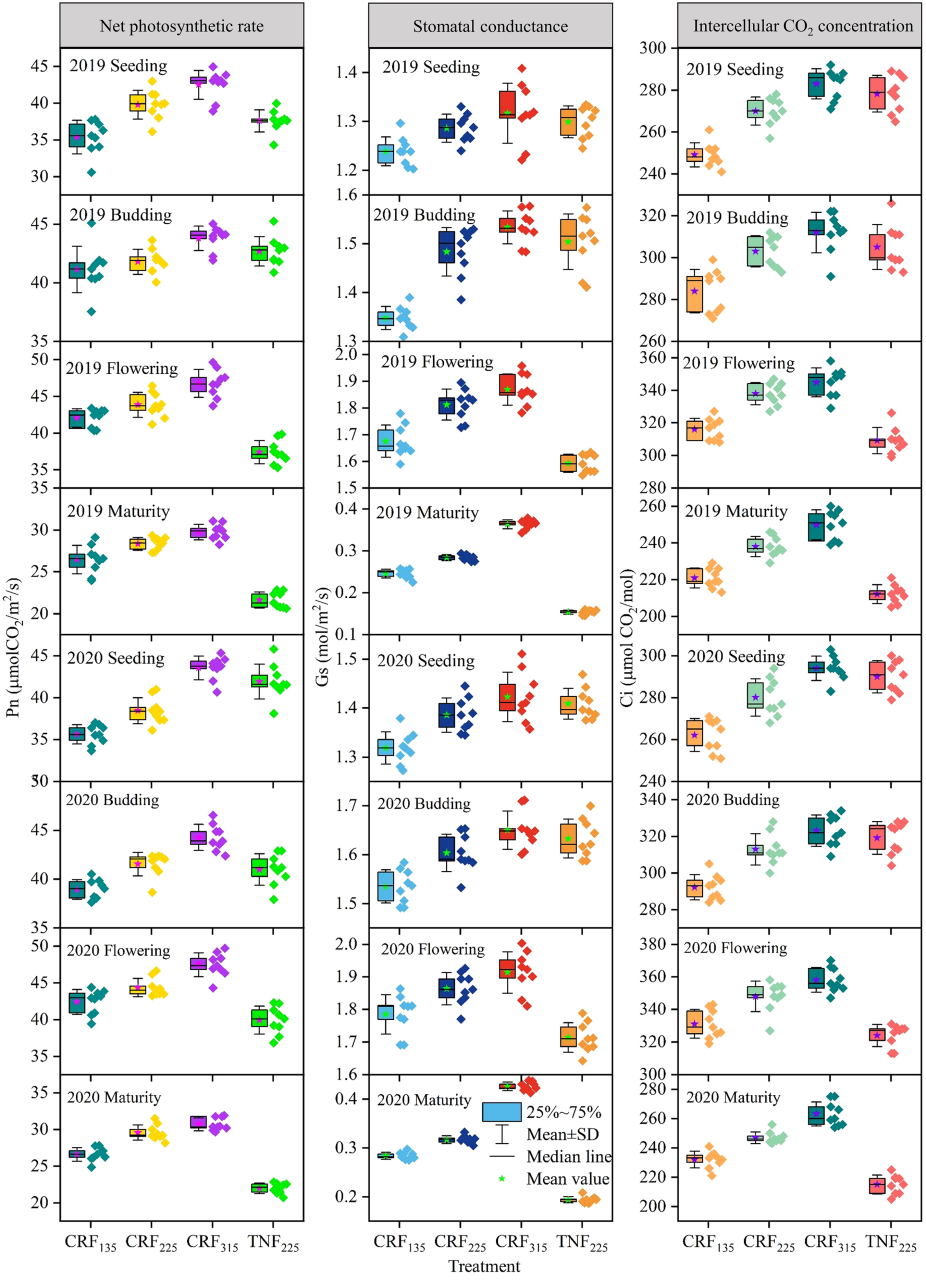
Variation trend of net photosynthetic rate (Pn, μmol CO_2_/m^2^/s), stomatal conductance (Gs, μmol H_2_O/m^2^/s) and intercellular CO_2_ concentration (Ci, μ mol CO_2_/mol) of sunflower under different treatments. CRF_135_, CRF_225_, CRF_315_ and TNF_225_ represent the controlled-release fertilizer (CRF) three nitrogen application rates (subscripts 135, 225, and 315 kg/ha) and traditional nitrogen fertilizer (TNF) considering one nitrogen application rate (subscripts 225 kg/ha).

The research data from 2019 and 2020 (**Figure 7**) exhibited the changes in the Pn, Gs, and Ci of sunflowers under different fertilization conditions. The results indicated that under the CRF treatment, increasing nitrogen application led to a gradual increase in all three indicators. At the seedling stage, compared with CRF_135_, the Pn for CRF_225_ and CRF_315_ increased by 10.25% and 21.27%, Gs by 4.48% and 7.13%, and Ci by 7.65% and 12.93%, respectively. By the budding stage, these increases were 4.15, 7.26, and 6.94% for CRF_225_ and 10.06%, 10.64%, and 10.24% for CRF_315_, respectively. The TNF_225_ treatment demonstrated the slightly higher Pn, Gs, and Ci than CRF with the same nitrogen amount, with Pn being 9.27% and 2.79% higher than CRF_225_ at the seedling and budding stages, respectively; Gs was 1.34% and 1.61% higher; and Ci was 3.27% and 1.29% higher. During the seedling and budding stages, Pn under TNF_225_ was 1.06% higher than that under CRF_225_.

By the flowering and maturity stages, the photosynthetic indicators under the TNF treatment had decreased to lower levels. At the maturity stage, except for the CRF_315_ treatment, all other treatments exhibited lower photosynthetic indicators. In particular, the Pn, Gs, and Ci of the CRF_315_ treatment were 15.02%, 31.29%, and 5.76% higher than those of the CRF_225_ treatment, respectively, whereas the CRF_225_ treatment showed decreases of 32.80%, 96.16%, and 13.56%, respectively, compared to TNF_225_.

### 3.5 Nitrogen accumulation and distribution

**Figure 8:**
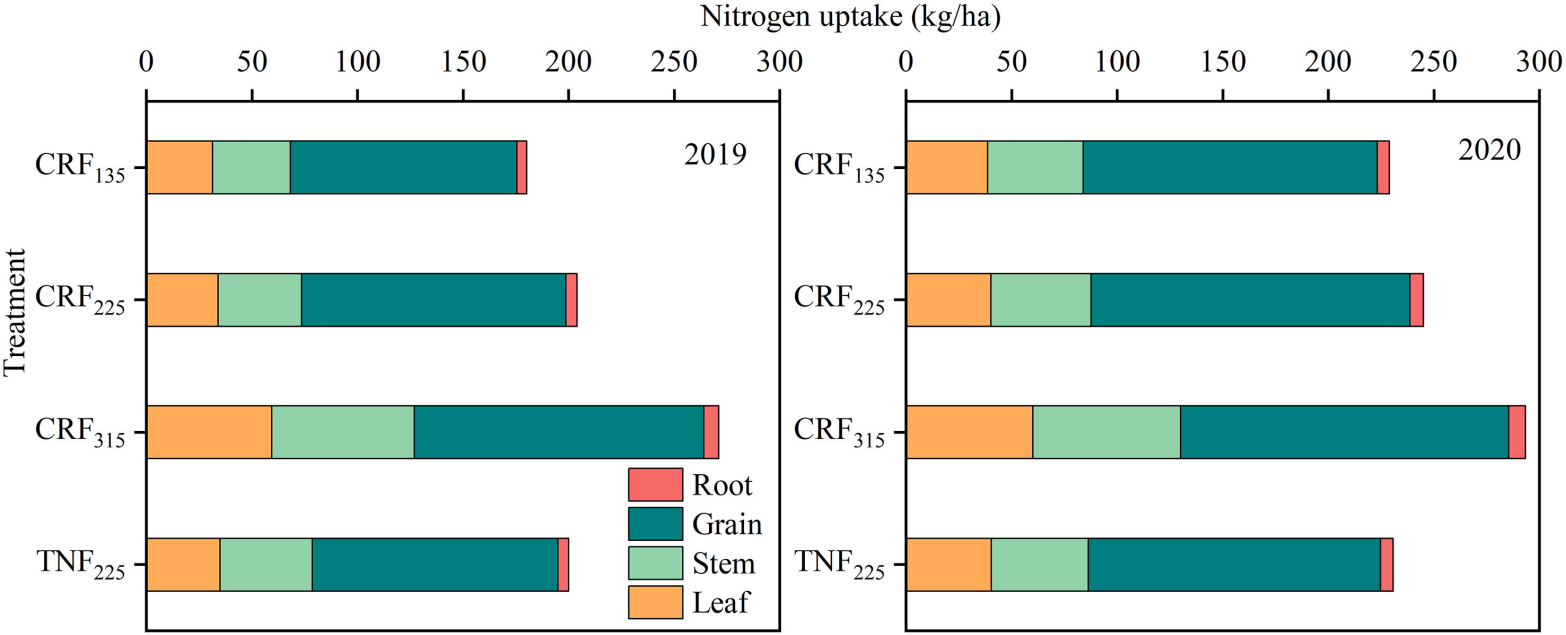
Nitrogen accumulation and distribution proportion in different organs at maturity stage of sunflower in 2019 and 2020 under the controlled-release fertilizer (CRF) three nitrogen application rates (subscripts 135, 225, and 315 kg/ha) and traditional nitrogen fertilizer (TNF) considering one nitrogen application rate (subscripts 225 kg/ha).

Throughout the sunflower growth period, significant differences in nitrogen accumulation in leaves, stems, seeds, roots, and total nitrogen at maturity were observed under different fertilization types and amounts (**Figure 8**). The total nitrogen accumulation increased by 10.17% and 39.33% for the CRF_225_ and CRF_315_ treatments, respectively, compared to CRF_135_, with CRF_225_ exhibiting a 4.17% increase over TNF_225_. As the nitrogen application rate increased under the CRF treatments, the nitrogen uptake by the leaves, stems, seeds, and roots also increased. When the application rate increased from 135 to 225 kg/ha, the nitrogen uptake by leaves, stems, seeds, and roots increased by 6.11%, 6.00%, 12.53%, and 15.06%, respectively. A further increase to 315 kg/ha resulted in significant increases in nitrogen uptake by leaves (61.75%), stems (59.91%), and roots (28.18%), whereas seed nitrogen uptake only rose by 6.10%. This suggested that higher nitrogen application primarily promoted the uptake in non-seed parts, with less impact on seed nitrogen uptake. The CRF treatments compared with TNF at the same nitrogen level increased the seed nitrogen uptake by 8.44%, indicating that CRF enhanced the nitrogen absorption by seeds more effectively. From the perspective of seed nitrogen uptake, the CRF application rate of 225 kg/ha was more suitable for the region.

### 3.6 Yield and nitrogen utilization efficiency

Previous research has presented that as the nitrogen application increased, the nitrogen uptake by sunflowers also increased, with the crops treated with CRF consistently absorbing more nitrogen than those treated with TNF under the same conditions. Similarly, increasing the nitrogen application led to higher hundred-seed weight, seed number, and yield of sunflowers (**Table 2**). Compared with the 135 kg/ha CRF treatment, the 225 kg/ha and 315 kg/ha CRF treatments increased the hundred-seed weight, seed number, and yield by 5.18%, 6.79%, and 14.87%, and by 6.46%, 9.50%, and 21.16%, respectively. However, increasing the CRF treatment from to 315 kg/ha only resulted in the modest increases of 1.22%, 2.54%, and 5.50% in these metrics, respectively, indicating diminishing returns. Additionally, the CRF treatments outperformed the TNF treatments in the hundred-seed weight, seed number, and yield, with the 225 kg/ha CRF treatment demonstrating the increases of 4.99%, 6.74%, and 11.84%, respectively, over TNF at the same rate. Therefore, applying 225 kg/ha of controlled-release fertilizer was optimal for sunflower cultivation in the region, effectively increasing the yield while promoting the efficient resource use.

**Table 2:** Yield, yield components, nitrogen harvest index (NHI), partial factor productivity (PFP) and nitrogen utilization efficiency (NUE) of sunflower applied with controlled-release fertilizer (CRF) three nitrogen application rates (subscripts 135, 225, and 315 kg/ha) and traditional nitrogen fertilizer (TNF) considering one nitrogen application rate (subscripts 225 kg/ha) in the 2019 and 2020 growing seasons.

Indicators	2019				2020			
	CRF_135_	CRF_225_	CRF_315_	TNF_225_	CRF_135_	CRF_225_	CRF_315_	TNF_225_
100-grain weight (g)	17.25 ± 0.58b	18.19 ± 0.37a	18.41 ± 0.8a	17.38 ± 0.53b	17.91 ± 0.42b	18.79 ± 0.65a	19.02 ± 0.63a	17.84 ± 0.64b
Number of grains	1035 ± 24.95b	1104 ± 24.6a	1136 ± 32.33	1029 ± 29.27b	1027 ± 29.43b	1098 ± 22.41a	1122 ± 38.43a	1034 ± 39.57b
Yield (kg/ha^2^)	3123.6 ± 250.83b	3649.2 ± 586.27a	3788.7 ± 152.77a	3459 ± 159.13ab	3258.15 ± 171.97b	3679.2 ± 378.75a	3943.5 ± 148.81a	3113.25 ± 301.07b
NHI (kg/kg)	0.61 ± 0.01b	0.63 ± 0.01a	0.52 ± 0.01c	0.6 ± 0.02b	0.62 ± 0.01ab	0.63 ± 0.01a	0.54 ± 0.01c	0.62 ± 0.01b
PFP (kg/kg)	23.14 ± 1.86a	16.22 ± 2.61b	12.03 ± 0.48c	15.37 ± 0.71b	24.13 ± 1.27a	16.35 ± 1.68b	12.52 ± 0.47c	13.84 ± 1.34c
NUE (kg/kg)	17.79 ± 1.43a	18.37 ± 2.95a	14.35 ± 0.58b	17.75 ± 0.82a	18.55 ± 0.98a	18.52 ± 1.91a	14.94 ± 0.56b	15.98 ± 1.54b

Under the CRF treatment, NHI and NUE initially increased and then decreased with the changes in nitrogen application, peaking at 225 kg/ha. The data from 2019 and 2020 indicated that compared to the CRF treatments of 135 and 315 kg/ha, the 225 kg/ha CRF treatment increased the average NHI by 2.24% and 18.90%, and NUE by 1.53% and 25.95%, respectively. However, PFP decreased as the nitrogen application increased, with the 135 kg/ha CRF treatment exhibiting the PFP 45.13% higher than the 225 kg/ha treatment, and the 225 kg/ha treatment presenting a PFP 32.73% higher than the 315 kg/ha treatment (**Table 2**).

Under the same nitrogen application conditions, the CRF treatments improved NHI, PFP, and NUE by 4.22%, 11.84%, and 9.70%, respectively, compared with the TNF treatments. These findings suggested that applying a moderate amount of nitrogen (225 kg/ha) with CRF optimized NHI and NUE in sunflowers while also enhancing PFP. Overall, CRF demonstrated a greater benefit than TNF in improving crop nitrogen utilization.

### 3.7 Correlation analysis among sunflower growth indicators

**Figure 9:**
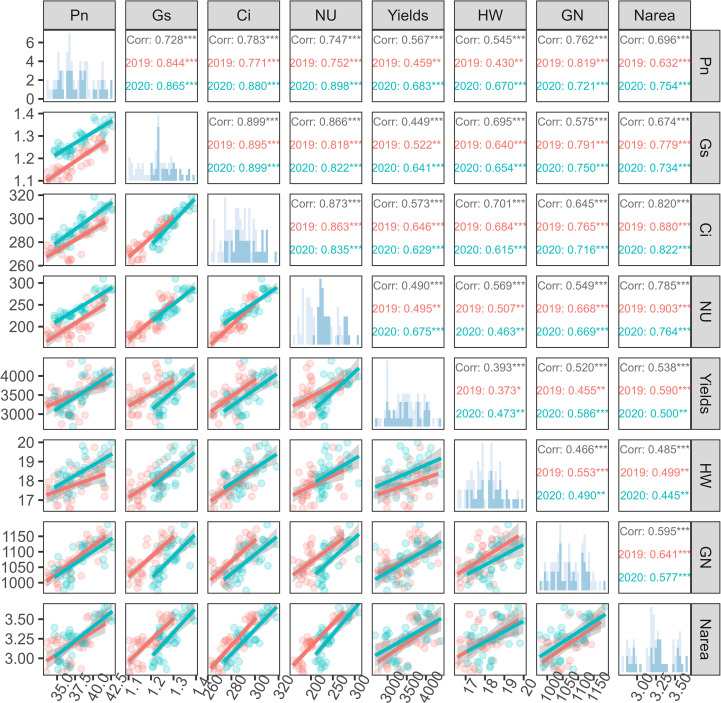
Pairwise correlations among net photosynthetic rate (Pn), stomatal conductance (Gs), intercellular CO_2_ concentration (Ci), nitrogen uptake (NU), grain yield, hundred-weight per plant (HW), grain number per plant (GN), and leaf nitrogen content per unit area (Narea) under different nitrogen treatments in the 2019 and 2020 growing seasons. The upper triangle shows Pearson correlation coefficients (***P < 0.001, **P < 0.01), with values separated by year (red for 2019, blue for 2020). The lower triangle presents corresponding scatter plots with linear regression lines. Histogram distributions are shown on the diagonal. Red and blue colors indicate data from 2019 and 2020, respectively. CRF treatments include three nitrogen application rates (135, 225, and 315 kg N/ha), and TNF refers to traditional nitrogen fertilizer applied at 225 kg N/ha.

During the 2019–2020 research period, the comprehensive analysis of the correlations between sunflower growth indicators was conducted, revealing their interrelationships. Using the Pearson’s correlation coefficient, significant correlations were identified among Pn, Gs, Ci, NU, yield, hundred-weight (HW), grain number (GN), and N_area_, whereas some relationships exhibited the variability (**Figure 9**).

A strong positive correlation was observed between Pn and Gs (2019: r = 0.844***; 2020: r = 0.865***), emphasizing the close link between the photosynthesis and stomatal behavior. The correlations between Ci and both Pn and Gs were also significant over the two years, reflecting a synergistic effect in the gas exchange process. Pn and NU indicated a significant positive correlation (2019: r = 0.752***; 2020: r = 0.898***), revealing a strong link between the photosynthetic activity and the crop nitrogen uptake, which was also influenced by environmental factors. The correlation between crop nitrogen uptake and yield (2019: r = 0.495***, 2020: r = 0.675***) suggested that nitrogen fertilization strategies may affect the yield potential differently each year. Overall, the sunflower yield demonstrated the significant positive correlations with other growth indicators, while the coefficients were around 0.5, indicating that the yield was affected by a variety of complex factors. Notably, the correlations of Ci and Pn with yield were above 0.5, highlighting the importance of photosynthesis and stomatal behavior in affecting crop yield.

## 4 Discussion

### 4.1 Response of leaf nitrogen distribution and photosynthetic parameters to nitrogen fertilizer type and application rate

Nitrogen distribution is a crucial factor influencing PNUE (Onoda et al., 2017; Zhuo et al., 2024), with different plant species exhibiting varied nitrogen allocations. The plants with higher PNUE allocated more nitrogen to the photosynthetic system and demonstrated higher growth rates (Poorter et al., 1990), whereas those with lower PNUE allocated more nitrogen to the non-photosynthetic systems (Onoda et al., 2004; van Ommen Kloeke et al., 2011). In this study, CRF improved the nitrogen allocation to the photosynthetic system compared to traditional fertilizers by providing a more stable and sustained nitrogen supply, thus enhancing PNUE. Specifically, under the 225 kg/ha CRF treatment, the nitrogen allocation to the photosynthetic system was greater than at other application levels, which promoted higher PNUE (**Figure 3**). Pn mainly relied on the light capture, electron transfer, and carboxylation, and nitrogen allocation to these processes (N_cl_, N_et_, and N_cb_, respectively) was positively correlated with PNUE (**Figure 4**). Therefore, increasing the proportions of N_cl_, N_et_, and N_cb_ was crucial for improving PNUE. Evolutionary algorithms have been used to simulate optimal nitrogen allocation in photosynthetic systems, suggesting that internal nitrogen redistribution may enhance photosynthetic capacity by up to 60% (Zhu et al., 2007). The nitrogen allocation to the photosynthetic system can be influenced by the plant growth environment. In cucumbers, as plants transition from high to low light intensity environments, the proportions of N_cb_ and N_et_ can decrease, while N_cl_ can increase (Trouwborst et al., 2011). Generally, most N_non-psn_ exist as N_store_, N_str_, and N_resp_, serving as a buffering mechanism for environmental adaptation (Cao et al., 2021). The species with higher proportions of N_non-psn_ exhibited greater tolerance to environmental stress (Onoda et al., 2004; van Ommen Kloeke et al., 2011). As the nitrogen application decreased, N_store_ supported the plant growth and development, and could be converted into N_psn_ to sustain photosynthesis (Liu et al., 2018b). Reducing the nitrogen application from 315 to 225 kg/ha increased the proportion of N_psn_, consistent with previous studies (Hou et al., 2019; Waring et al., 2023). This study also highlighted the significant impact of CRF on sunflower photosynthetic parameters (Pn, Gs, and Ci), indicating that these parameters could be optimized by adjusting the nitrogen fertilizer application rate (**Figure 5**). These results highlighted the critical role of nitrogen management strategies in promoting the crop photosynthetic efficiency. By providing a more stable and sustained nitrogen supply, CRF enhanced the leaf physiological states and the stomatal functions, thereby improving Pn and Gs. These findings aligned with the existing literature on the positive effects of nitrogen supply on crop growth and photosynthesis (Hong et al., 2022; Mu and Chen, 2021; Vos et al., 2005), demonstrating the complex interaction between nitrogen supply and plant photosynthetic mechanisms. Nitrogen is essential for synthesizing the chlorophyll and photosynthetic enzymes (Ali et al., 2019; Evans, 1989; Evans and Clarke, 2019), which are crucial for photosynthesis. Therefore, enhancing the nitrogen supply, particularly through the sustained and stable provision achieved with CRF, may boost chlorophyll synthesis and increase photosynthetic enzyme activity, thereby directly improving Pn. Additionally, an adequate nitrogen supply enhances plant stomatal regulation capabilities (Radin et al., 1982), as evidenced by increased stomatal conductance (Gs), which facilitated more efficient carbon dioxide absorption and further promoted the photosynthesis.

The study results demonstrated that the CRF treatment outperformed the TNF treatment in photosynthetic performance during key growth stages, indicating the importance of optimizing the nitrogen supply during critical crop growth periods. This advantage was attributed to the slow-release characteristic of CRF, which could provide an appropriate nitrogen supply when crop demand was the highest, thereby supporting the optimal growth and development (Shaviv, 2001; Zhang et al., 2024). This finding demonstrated the need to align the nitrogen management strategies with the temporal requirements of crop growth and development, which could have significant implications for improving the nitrogen fertilizer use efficiency and reducing the environmental impact. Selecting the appropriate type and amount of nitrogen fertilizer could significantly enhance the crop photosynthetic efficiency, increase yield, and mitigate the environmental impacts of nitrogen fertilizers.

### 4.2 Nitrogen accumulation, distribution, and utilization efficiency

The results of this study revealed the significant differences in the nitrogen accumulation and distribution at various sunflower growth stages under different CRF treatments, with the 225 kg/ha application rate achieving the optimal nitrogen accumulation and distribution in the leaves, stems, seeds, and roots (**Figure 8**). This finding demonstrated the complex interaction between the nitrogen supply and the crop physiological responses (Meng et al., 2021; Pan et al., 2019; Rurinda et al., 2020), highlighting the need for precise nitrogen application management to optimize the crop growth and enhance NUE (Oliveira et al., 2025; Raghuram et al., 2022; Yang et al., 2022). The continuous and stable nitrogen supply provided by CRF promoted the efficient nitrogen absorption and utilization, thereby affecting the nitrogen distribution pattern within the plant. Compared with TNF, CRF reduced the nitrogen accumulation in non-target parts, such as leaves and stems, and increased its transfer to seeds, which was crucial for improving the crop yield and quality. This optimized nitrogen distribution was linked to CRF’s enhancement of nitrogen assimilation and transport mechanisms by CRF, reflecting the adaptive response of the crop to the nitrogen fertilizer management strategies.

Improving NUE can be a key goal in the nitrogen fertilizer management for sustainable agricultural development (Ren et al., 2022a; Shi et al., 2024). This study discovered that under the CRF treatment, the sunflower NUE increased with the application rate to the peak of 225 kg/ha before declining, indicating that this rate provides the optimal NUE. This finding highlighted the importance of identifying an appropriate nitrogen application amount that met the crop growth needs without leading to excessive nitrogen accumulation or loss, which was essential for optimizing the nitrogen use and minimizing the environmental impact. Further analysis revealed that CRF, compared to TNF, more effectively enhanced the crop PNUE and NHI, demonstrating its superior ability to improve the direct nitrogen utilization efficiency and convert nitrogen into yield (**Table 2**). Although CRF_315_ resulted in higher biomass and grain yield, it also caused excessive nitrogen accumulation in vegetative tissues (i.e., leaves and stems), which led to a lower NHI and reduced NUE (Hu et al., 2023b; Li et al., 2022). In contrast, CRF_225_ achieved a more balanced nitrogen distribution between vegetative and reproductive organs, thereby contributing to improved NUE. This efficacy was attributed to the ability of CRF to reduce the nitrogen fluctuations and provide a more stable nitrogen source (Ali et al., 2024; Geng et al., 2015; Li et al., 2017; Trenkel, 2021), indicating the potential benefits of controlled-release technology in agricultural production.

The absorption, transformation, and distribution of nitrogen within plants involve complex biochemical reactions, including the regulation of nitrogen assimilation and transport protein expression (Tegeder and Masclaux-Daubresse, 2018; Xu et al., 2012). CRF may enhance the nitrogen utilization efficiency by influencing these biochemical pathways. For instance, a stable nitrogen supply could prompt plants to adjust their nitrogen assimilation enzyme activities (Xiong et al., 2021), improve the nitrogen fixation in organic forms, and increase the nitrogen availability during critical growth stages (Liu et al., 2022; Sharma et al., 2021). Future research should investigate how CRF specifically affects these biochemical processes and signaling pathways and how these effects interact with crop growth traits and yield performance. The practical optimization of nitrogen fertilizer management should not only aim to increase yield but also balance crop quality, resource use efficiency, and environmental impact. By precisely controlling the timing and amount of nitrogen supply, it is possible to maximize the crop nutritional value and economic return while minimizing the environmental burden. Additionally, considering the crop varieties, soil conditions, and climate change responses can further enhance the efficiency and sustainability of N management. Therefore, future efforts should integrate new nitrogen fertilizer technologies, such as CRF, with crop physiology research, precision agriculture, and environmental protection measures to promote comprehensive sustainable agricultural development.

## 5 Conclusion

This study comprehensively evaluated the effects of CRF application on the spatiotemporal distribution of soil NO_3_-N throughout the crop growth period and its subsequent effects on nitrogen distribution in crop leaves, photosynthetic characteristics, nitrogen accumulation and distribution, yield and its components, PNUE, and NUE. These findings demonstrated the significant benefits of CRF in optimizing the nitrogen management, enhancing the crop production efficiency, and promoting the environmental sustainability. Specifically, CRF maintained the stable concentration of NO_3_-N in the soil during crop growth, improved the proportion of N_psn_ in leaves, enhanced photosynthetic efficiency, and optimized the nitrogen accumulation and distribution within the plant, particularly at an application rate of 225 kg/ha. Moreover, CRF significantly improved NUE compared with TNF. These results proved the value of CRF and precise management strategies for enhancing the crop photosynthesis, yield, and NUE by altering the nitrogen distribution in crop leaves, thereby achieving the efficient and sustainable agricultural production. Future research should further investigate the CRF application effects on various crops and under different environmental conditions and evaluate the long-term impacts of nitrogen fertilizer management on agricultural ecosystem services.

## Data Availability

The raw data supporting the conclusions of this article will be made available by the authors, without undue reservation.
